# Enhanced spatial models for predicting the geographic distributions of tick-borne pathogens

**DOI:** 10.1186/1476-072X-7-15

**Published:** 2008-04-15

**Authors:** Michael C Wimberly, Adam D Baer, Michael J Yabsley

**Affiliations:** 1 Geographic Information Science Center of Excellence, South Dakota State University, Brookings, SD, USA; 2 Southeastern Cooperative Wildlife Disease Study, University of Georgia, Athens, GA, USA; 3 Warnell School of Forestry and Natural Resources, University of Georgia, Athens, GA, USA

## Abstract

**Background:**

Disease maps are used increasingly in the health sciences, with applications ranging from the diagnosis of individual cases to regional and global assessments of public health. However, data on the distributions of emerging infectious diseases are often available from only a limited number of samples. We compared several spatial modelling approaches for predicting the geographic distributions of two tick-borne pathogens: *Ehrlichia chaffeensis*, the causative agent of human monocytotropic ehrlichiosis, and *Anaplasma phagocytophilum*, the causative agent of human granulocytotropic anaplasmosis. These approaches extended environmental modelling based on logistic regression by incorporating both spatial autocorrelation (the tendency for pathogen distributions to be clustered in space) and spatial heterogeneity (the potential for environmental relationships to vary spatially).

**Results:**

Incorporating either spatial autocorrelation or spatial heterogeneity resulted in substantial improvements over the standard logistic regression model. For *E. chaffeensis*, which was common within the boundaries of its geographic range and had a highly clustered distribution, the model based only on spatial autocorrelation was most accurate. For *A. phagocytophilum*, which has a more complex zoonotic cycle and a comparatively weak spatial pattern, the model that incorporated both spatial autocorrelation and spatially heterogeneous relationships with environmental variables was most accurate.

**Conclusion:**

Spatial autocorrelation can improve the accuracy of predictive disease risk models by incorporating spatial patterns as a proxy for unmeasured environmental variables and spatial processes. Spatial heterogeneity can also improve prediction accuracy by accounting for unique ecological conditions in different regions that affect the relative importance of environmental drivers on disease risk.

## Background

Maps of disease risk have a broad spectrum of applications in the health sciences. Disease maps can aid the diagnosis of individual cases by providing information about the likelihood of exposure to specific infectious agents [1]. Disease maps are also frequently used in regional assessments of public health. Spatial patterns of disease risk can be combined with other geographic datasets to identify and evaluate populations at risk [2], and to aid in predicting future disease outbreaks and epidemics [3,4]. Although disease risk is defined as the probability of an individual contracting a disease within a specific time period [5], direct measurements of risk can be difficult to obtain, and disease maps are often based on presumed correlates of risk such as vector abundance, pathogen prevalence in a sentinel species, or disease frequency in human populations. Another challenge in developing disease maps is that the underlying data may be available at a limited number of isolated locations. This problem can be particularly acute for emerging infectious diseases, which are likely to be misdiagnosed and underreported, and in developing countries where surveillance may be limited or nonexistent. Therefore, it is often necessary to interpolate between isolated sample locations to generate a continuous surface of disease risk predictions.

One solution to this problem is to model disease risk as a function of one or more environmental variables. This approach is based on the assumption that the environment influences development and transmission of pathogens, habitats for disease vectors and hosts, or human exposure to pathogens. To be used in disease mapping, environmental data must be available as complete spatial coverages that allow model calibration at sites where disease data exist, and model-based predictions at other locations where disease data are unavailable. Climate is recognized as a major constraint on the geographic ranges of infectious diseases, and interpolated climate datasets have been used to predict the distributions of tick vectors in the United States [6], Europe [7], and southern Africa [8]. Spatial variability in land cover, soils, and geology also affect habitat suitability for vector species, and these variables have been used to predict the spatial pattern of habitat suitability for *Ixodes scapularis *in the north-central United States [9]. Spectral indices derived from satellite imagery provide information about environmental characteristics such as vegetation cover, moisture, and temperature, and have been used to develop disease risk maps ranging from landscape patterns of tick habitat suitability [10] to the distribution of malaria across Africa [11].

Spatial autocorrelation is an important statistical consideration in the development of predictive models of disease risk. Sites located close to one another tend to have similar disease risk because they share similar environments and are connected via communicable disease spread or vector and host dispersal. Ordinary least squares regression, generalized linear models, and other standard statistical modelling methods assume that any spatial pattern in the response variable can be entirely explained by the set of predictor variables, and that model residuals are independent and identically distributed [5]. Problems with spatial autocorrelation can arise when there are relevant environmental predictors that have not been included in the model, or when disease patterns are affected by dispersal limitations as well as the environment. Failure to fully account for spatial autocorrelation results in biased estimates of the coefficients and their standard errors, which in turn affect model predictions and statistical tests on the coefficients [12].

Despite these challenges, spatial autocorrelation also presents opportunities for improving model predictions when the association between disease risk and the available environmental data is weak. Put simply, if disease risk exhibits some degree of spatial clustering, a location surrounded by sites with high disease risk would be expected to have a high disease risk, and a location surrounded by sites with low disease risk would be expected to have a low disease risk. Spatial interpolation based on associations with neighbouring sites can be implemented using a variety of statistical techniques. A study of the tick-borne pathogen *Ehrlichia chaffeensis *in the southern U.S. found that spatial interpolation based on indicator kriging outperformed logistic regression models based on environmental variables [13]. Predictive mapping studies of tick distributions have applied methods such as co-kriging [14], and autologistic regression [6] to combine information about environmental relationships with spatial autocorrelation in a predictive framework.

Another consideration in developing disease risk models is the phenomenon of spatial heterogeneity [15] (also referred to as spatial non-stationarity [16]), which occurs when the influences of environmental variables on disease risk are not uniform across the region of interest. For example, sub-regional logistic regression models provided evidence of geographically varying environmental constraints on the distribution of *E. chaffeensis *and yielded more accurate predictions of pathogen presence than a single model fitted for the entire region [13]. Similarly, the relationship between climate and the distribution of *Ixodes ricinus *in Europe was found to vary across ecoregions [7]. Statistical techniques such as geographically weighted regression (GWR) [16] have been developed specifically to analyze the spatial variability of regression parameters, but have only recently been applied to analyze spatial patterns of disease risk [17-19]. The implication for spatial modelling is that if there is indeed spatial variability in the relationships between disease risk and environmental variables, models that explicitly account for this heterogeneity are likely to yield more accurate predictions

This study compared alternative methods for developing predictive maps of the geographic distributions of two tick-borne pathogens in the southern United States. *Ehrlichia chaffeensis*, the causative agent of human monocytotropic ehrlichiosis, is transmitted by *Amblyomma americanum *(lone-star tick). *Anaplasma phagocytophilum*, the causative agent of human granulocytotropic anaplasmosis (previously called HGE agent), is transmitted by *Ixodes scapularis *(black-legged tick). *E. chaffeensis *is maintained in a zoonotic cycle that includes white-tailed deer (*Odocoileus virgnianus*) as a keystone host for larval, nymph, and adult *A. americanum *[20] and the primary reservoir for *E. chaffeensis *[21]. In contrast, *A. phagocytophilum *is maintained in a zoonotic cycle in which white-tailed deer are a primary hosts for adult *I. scapularis*, but additional bird and mammal species are required to serve as hosts for the larval and nymph stages [22]. The white-footed mouse, *Peromyscus leucopus*, is a particularly important host for immature *I. scapularis *in the eastern United States and is also a competent reservoir for *A. phagocytophilum *[23]. In general, *A. americanum *is more tolerant of desiccation than *I. scapularis *and can occupy more exposed microsites and remain active at lower humidity [24,25].

Although a variety of methods have been proposed for improving predictive spatial models by incorporating spatial autocorrelation or spatial heterogeneity into environmental models, there have been no comparative assessments of the accuracy that is gained by applying these more complex approaches in disease risk mapping. The main goal of this research was to determine whether incorporating spatial autocorrelation and spatial heterogeneity would improve environmental predictions of the geographic distributions of *E. chaffeensis *and *A. phagocytophilum*. A further goal was to determine whether the modelling strategies that were most effective for each pathogen reflected differences in the underlying host relationships and vector ecology.

## Methods

### Serology Data

**Figure 1:**
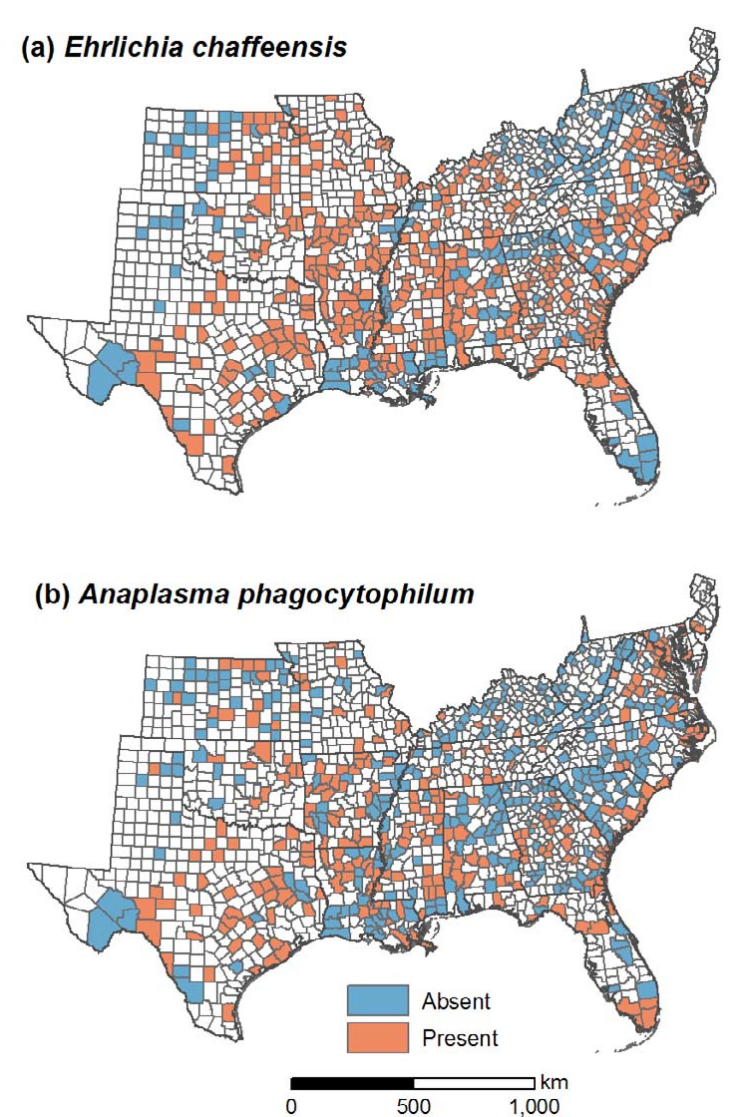
**Presence/absence of *Ehrlichia chaffeensis *and *Anaplasma phagocytophilum *in the southeastern United States based on serology of white-tailed deer herds**.

Data on the county level distributions of *E. chaffeensis *and *A. phagocytophilum *were available from previous research on their serostatus in *Odocoileus virginianus *(white-tailed deer) populations [26,27]. This surveillance approach was based on immunofluorescent antibody (IFA) tests performed on serum samples from white-tailed deer, and its efficacy has been confirmed by comparisons with polymerase chain reaction assays and culture isolations. *E. chaffeensis *and *A. phagocytophilum *were each sampled from 567 white-tailed deer populations distributed across 18 states. Serological data for each population were georeferenced by county. *E. chaffeensis *and *A. phagocytophilum *were classified as present in counties where one or more deer had antibodies reactive to the pathogen (Figure 1).

A descriptive analysis was carried out to quantify differences in the spatial patterns of these pathogens. Indicator semivariograms [28] were computed to characterize spatial autocorrelation for *E. chaffeensis *and *A. phagocytophilum*. The spatial location of each county was represented by its centroid, and presence (1) or absence (0) of the pathogen in each county was used as the indicator variable.

### Environmental Data

Environmental variables characterizing climate, land cover, and host populations within each county were obtained from a variety of sources. Climate variables were computed from 1-km Daymet grids which summarized monthly minimum temperature, maximum temperature, and precipitation over the period 1980–97 [29]. Monthly relative humidity was computed using estimates of ambient and saturation vapor pressure derived from monthly minimum and maximum temperatures [30]. Land-cover variables were derived from the National Land Cover Dataset, which was created using 30-m resolution Landsat imagery collected in 1992 [31]. These data were used to compute the proportion of each county covered by forests, which included evergreen, deciduous and mixed forest as well as forested wetlands. The spatial pattern of forest cover within each county was characterized using a fragmentation index, which quantified the frequency of edges between forest and human land-use pixels (e.g. urban, agriculture) relative to the frequency of adjacent forested pixels [32]. Deer density data from 1999 were obtained as a paper map from the Quality Deer Management Association (Watkinsville, GA, USA). Deer density was mapped as an index with five levels: (1) deer absent, rare or urban with unknown population; (2) < 15 deer/km^2 ^; (3) 15–30 deer/km^2 ^; (4) 30–45 deer/km^2^; and (5) > 45 deer/km^2^. The map was digitized, georeferenced and converted to a 1-km grid. Deer density was summarized for each county as the density index that characterized the majority of the county.

**Figure 2:**
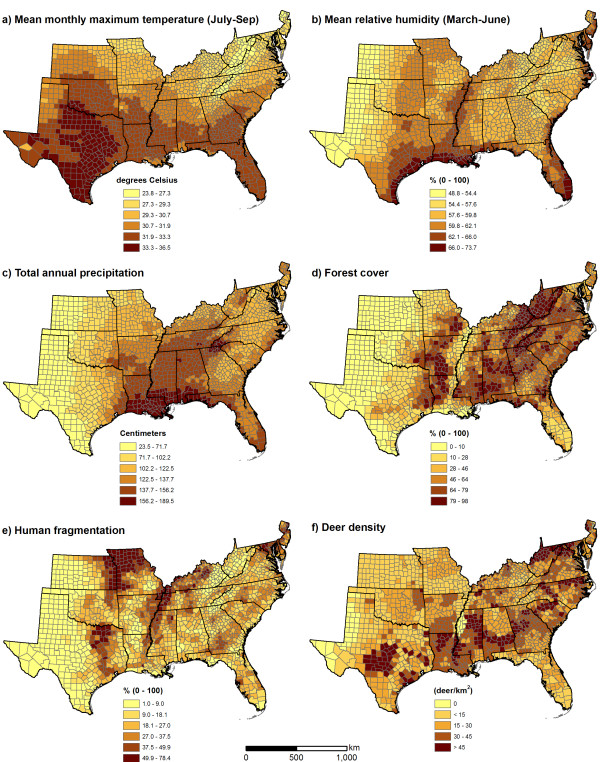
Predictor variables used to develop environmental models of *Ehrlichia chaffeensis *and *Anaplasma phagocytophilum *in the southeastern United States.

A set of predictor variables was previously chosen for each pathogen through a multi-model comparison exercise [18], and these variables were used to develop the environmental models considered in this study (Figure 2). July-September mean maximum monthly temperature, March-June mean monthly humidity, annual precipitation, and percent forested land cover were used as environmental predictors for both *E. chaffeensis *and *A. phagocytophilum*. In addition, the fragmentation index was used as a predictor variable for *E. chaffeensis*, and deer density was used as a predictor variable for *A. phagocytophilum*.

**Figure 3:**
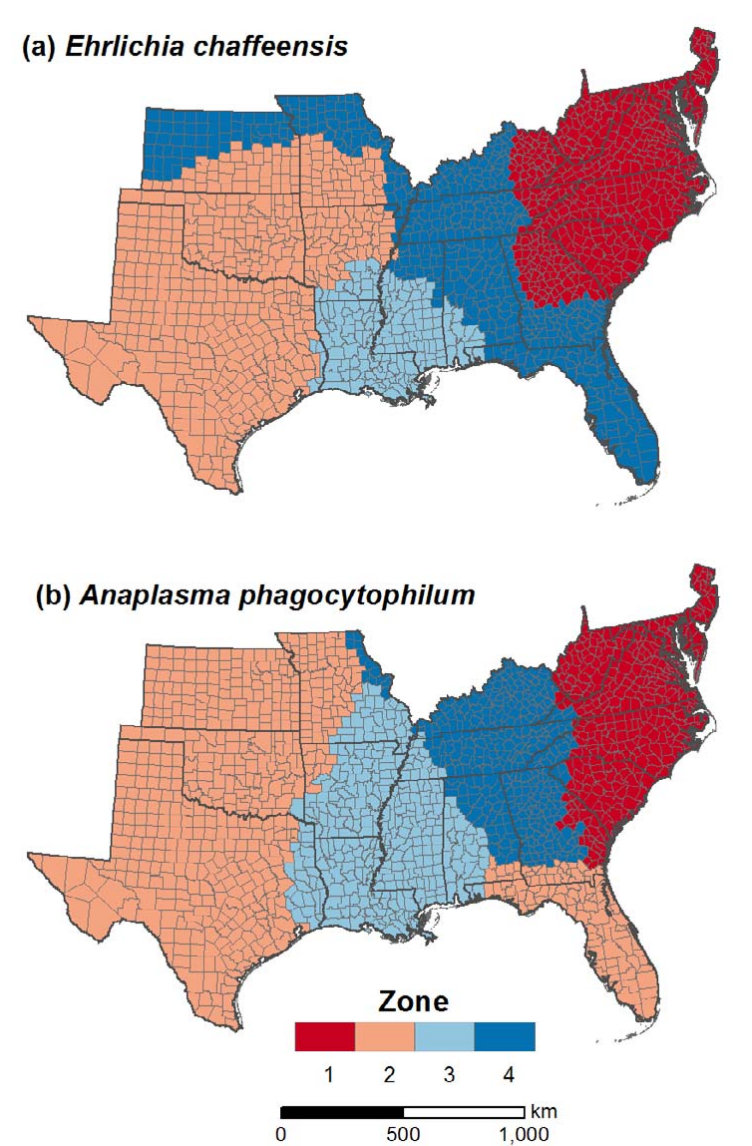
**Geographic zones of the southeastern United States used in the development of the local environmental models.** The zones were derived in a previous study using *k*-means clustering of geographically weighted regression results [18].

Geographic zones were previously identified to characterize spatial heterogeneity in the influences of environmental variables on the distributions of *E. chaffeensis *and *A. phagocytophilum*. The zones were created via *k*-means clustering of the results of a geographically weighted regression (GWR) analysis of pathogen distributions, as documented in a previous study [18]. GWR produces local estimates of regression coefficients for each sample location [16]. Each cluster thus delineates an area in which pathogen-environment relationships are relatively homogeneous, but distinctive from the other clusters. This method identified a set of four geographic zones for each pathogen (Figure 3). Although the underlying GWR models and the resulting geographic zones were different for *E. chaffeensis *and *A. phagocytophilum*, both pathogens exhibited a general shift from climatic constraints in the southeastern U.S. to land cover and deer density constraints in the south-central U.S. [18].

### Statistical Models

We used a hierarchical Bayesian modelling approach to fit statistical models of pathogen presence/absence at the county level. We chose this technique because it allowed us to examine environmental correlates, spatial autocorrelation, and spatial heterogeneity in a consistent statistical framework. The binary response variable, *Y*_*i*_, denoting pathogen presence/absence in each county was assumed to follow a Bernoulli distribution *Y*_*i *_~ Bernoulli(*p*_*i*_)

where *p*_*i *_was the probability of pathogen presence in county *i*, hereafter referred to as the *endemicity probability *[26]. The probability of pathogen presence was in turn modelled as a function of predictor variables characterizing local environmental characteristics and spatial association with neighbouring counties. The following set of five alternative models was considered.

(1) The *global environmental model *predicted *p*_*i *_as a function of co-located environmental variables. In the global model, a single parameter was fitted to quantify the influence of each environmental variable across the entire study area.

$$\text{logit}(p_{i})=\log\left[p_{i}/(1-p_{i})\right]=b_{0}+\sum_{j=1}^{v}b_{j}x_{ij}$$

where *j *indexed *v *explanatory variables, *b*_0 _was the intercept, *b*_*j *_were the parameters, and *x*_*ij *_were the environmental variables.

(2) The *local environmental model *also predicted *p*_*i *_as a function of co-located environmental variables. To account for spatial heterogeneity, multiple parameters were fitted for each environmental variable to account for spatial heterogeneity in environmental effects across geographic zones (Figure 3).

$$\text{logit}(p_{i})=b_{00}+\sum_{j=1}^{v}b_{j0}x_{ij}+\sum_{k=1}^{s-1}b_{0k}z_{k}+\sum_{j=1}^{v}\sum_{k=1}^{s-1}b_{jk}z_{k}x_{ij}$$

where *k *indexed geographic zones, *s* was the number of geographic zones, *b*_00_ was the intercept for the baseline zone, *b*_*j*0 _were the parameters for the baseline zone, *z*_*k *_were indicator variables for the *s*-1 other zones, *b*_0*k *_were the deviations of the intercept in zone *k *from *b*_00_, and *b*_*jk *_were the deviations of the parameter for environmental variable *j *in zone *k *from *b*_*j*0_. Zone 1 was used as the baseline zone in all models (Figure 3).

(3) The *spatial autoregressive *model predicted *p_i _*as a function of endemicity in neighbouring counties

logit(*p*_*i*_) = *b*_0 _+ *ρ*_*i*_

where *ρ*_*i *_was a spatial random effect that was modelled as a conditionally autoregressive (CAR) process. These random effects adjusted the endemicity probability up or down depending on the values of *ρ*_*i *_in surrounding counties [33].

(4) The *global environmental-autoregressive *model was a combination of models (1) and (3).

$$\text{logit}(p_{i})=b_{0}+\sum_{j=1}^{v}b_{j}x_{ij}+\rho_{i}$$

(5) The *local environmental-autoregressive *model was a combination of models (2) and (3).

$$\text{logit}(p_{i})=b_{00}+\sum_{j=1}^{v}b_{j0}x_{ij}+\sum_{k=1}^{s-1}b_{0k}z_{k}+\sum_{j=1}^{v}\sum_{k=1}^{s-1}b_{jk}z_{k}x_{ij}+\rho_{i}$$

Models were fitted via Markov Chain Monte Carlo (MCMC) simulation using WinBUGS software [34]. Vague prior distributions for the environmental parameters were specified as *b*_*j *_~ *b*_*jk *_~ *N*(0, 10^6^). The spatial random effect was modelled as a conditional autoregressive (CAR) process in which the distribution of each spatial effect had a Gaussian distribution centred on the mean of the neighbouring values.

$$\rho_{i}|\rho_{i\neq j}\sim N\left(\frac{\sum_{i\neq j}w_{ij}\rho_{i}}{\sum_{i\neq j}w_{ij}},\frac{\sigma_{\rho }^{2}}{\sum_{i\neq j}w_{ij}}\right)$$

where *w*_*ij *_were the neighbourhood weights and *σ*^2^_*ρ *_was a hyperparameter specifying the prior variance of the spatial random effects. The *w*_*ij *_were specified based on a queen's adjacency rule, in which counties sharing a common boundary were considered neighbours. In spatial Bayesian models, a hyperprior for 1/*σ*^2^_*ρ *_is commonly specified as a gamma distribution such as Γ(0.001, 0.001) or Γ(0.5, 0.0005) [35]. However, in the present application these specifications led to difficulties with MCMC convergence. Instead, we specified a truncated normal hyperprior for *σ*^2^_*ρ*_, which has been suggested as one alternative to the gamma distribution [36]. We used a moderately informative specification of *σ*^2^_*ρ *_~ *N*(0, 10), truncated at zero so that *σ*^2^_*ρ *_was always positive. Sensitivity analyses using alternative specifications of *σ*^2^_*ρ *_~ *N*(0, 5) and *σ*^2^_*ρ *_~ *N*(0, 20) yielded similar parameter estimates and prediction accuracies, demonstrating that our results were robust to changes in the specification of *σ*^2^_*ρ*_. Flat priors were used for the intercepts *b*_*0 *_and *b*_*00*_.

The data used to fit the models and generate predictions included *Y*_*i *_values for the counties with serology data, along with *x *and *z *values for all of the counties in the study area. Initial values were specified for all model parameters, including the coefficients for each environmental variable and the spatial random effects for each county. The posterior values of these parameters were updated during each step of the MCMC algorithm, and the parameters were then used to compute values of *p*_*i *_for all counties. Convergence of the MCMC algorithm was evaluated through visual examination of the trace plots and through Brooks-Rubin-Gelman diagnostic plots [37]. Based on these evaluations, a burn in of 20,000 steps was sufficient to achieve convergence for all models, and the posterior parameters values were sampled at 20,000 additional steps. The endemicity probability for each county was computed as the mean of *p*_*i *_across the 20,000 steps.

### Model Evaluation

Cross-validation was used to compare model performance at predicting pathogen presence in unsampled counties. The 567 counties with serological data were randomly split into four subsets of approximately equal size, and four WinBUGS runs were carried out for each model. In each of these runs, one of the four subsets was set aside for model evaluation, and the remaining three subsets were used to fit the model.

The predictive capabilities of the models were evaluated by computing the area under the receiver operating characteristic curve (AUC) for each model. The receiver operating characteristic curve describes relationship between the true positive rate and the false positive rate using a range of thresholds to classify pathogen presence and absence based on *p*_*i *_[38]. The AUC statistic can be interpreted as the probability that a randomly selected county where the pathogen is present will have a higher *p*_*i *_value than a randomly selected county where the pathogen is absent. We also selected an optimal classification threshold for each model by computing classification accuracy (percent of counties correctly classified) for a range of thresholds and choosing the threshold with the highest accuracy. Sensitivity (percent of positive counties correctly classified) and specificity (percent of negative counties correctly classified) were also computed using this optimal threshold.

Maps of the predicted distributions of each pathogen were generated by plotting the spatial distribution of predicted *p*_*i *_values for each of the five models. To generate these maps, models were fitted using pathogen data from all 567 counties with serology data to utilize all the available information and generate the best possible spatial predictions. Pathogen presence/absence data from the serology database were overlaid on the predicted endemicity probabilities to visually assess spatial patterns of prediction accuracy for the various models

## Results

**Figure 4:**
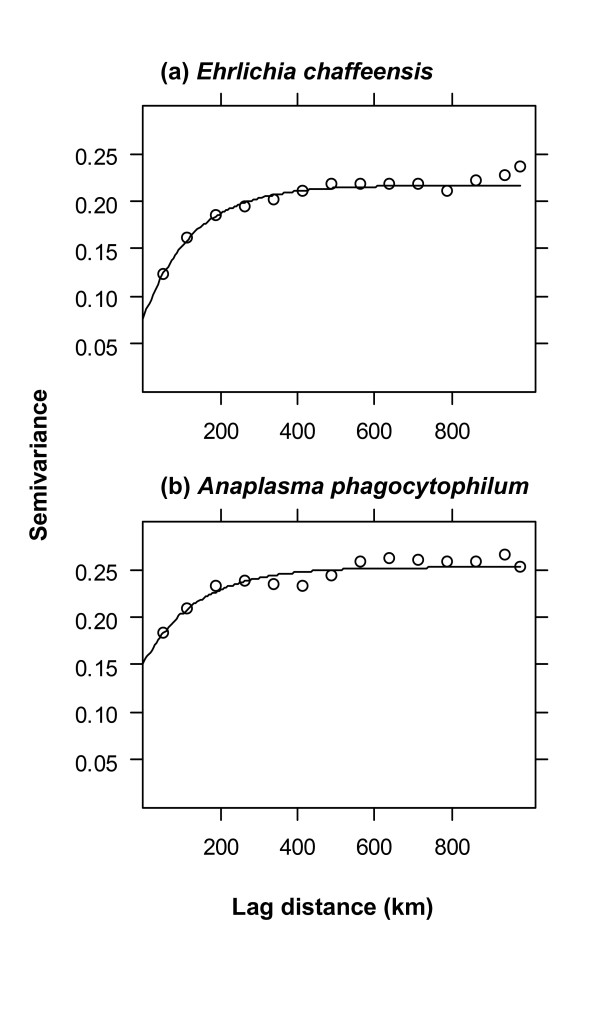
Indicator semivariograms (1 = present, 0 = absent) of the geographic distributions of *Ehrlichia chaffeensis *and *Anaplasma phagocytophilum*.

Semivariograms were computed for each pathogen using a bin width of 75 km (Figure 4). Exponential models were fitted to the semivariograms to quantify both the strength and scale of spatial autocorrelation (Table 1). The higher normalized sill for *E. chaffeensis *indicated that a larger portion of the variability in the distribution of this pathogen was spatially structured. In contrast, the lower normalized sill for *A. phagocytophilum *indicated that this pathogen had a relatively weak spatial pattern with a large random component. The ranges for the two pathogens were similar, indicating little difference in the scale of spatial autocorrelation.

**Table 1:** Parameters for exponential models fitted to indicator semivariograms of the distributions of two tick-borne pathogens

Pathogen	Range (*a*)	Nugget (*c*_*0*_)	Partial Sill (*c*_*1*_)	Normalized sill
*Ehrlichia chaffeensis*	128.7 km	0.0767	0.140	0.646
*Anaplasma phagocytophilum*	137.3 km	0.151	0.102	0.402

Total sill is the maximum semivariance, *c*_*0 *_+ *c*_*1*_.

Normalized sill is the ratio of the partial sill to the total sill, *c*_*1*_/(*c*_*0 *_+ *c*_*1*_)

Both pathogens had positive relationships with temperature, humidity, and forest cover and negative relationships with precipitation. In addition, *E. chaffeensis *had a positive relationship with the fragmentation index, and *A. phagocytophilum *had a positive relationship with deer density. However, these relationships varied considerably among geographic zones in the local environmental and local environmental-autoregressive models (see Additional file 1).

For *E. chaffeensis*, the AUC for the global environmental model was lower than all the other models (Table 2). The local environmental model that incorporated spatial heterogeneity had a higher AUC than the global environmental model. The spatial autoregressive model had the highest AUC of all the *E. chaffeensis *models, although the global environmental-autoregressive and local environmental-autoregressive models were only slightly lower. The ranking of *E. chaffeensis *models based on classification accuracy was the same as the ranking based on AUC. Predictions of *E. chaffeensis *presence/absence had high sensitivity (> 0.9) and comparatively low specificity (< 0.6) for all the models tested.

**Table 2:** Predictive accuracy of five statistical models for the distribution of *Ehrlichia chaffeensis *in the southeastern and south-central United States.

Model	AUC	Accuracy	Sensitivity	Specificity	Threshold
Global environmental	0.745	0.776	0.905	0.497	0.555
Local environmental	0.801	0.801	0.905	0.575	0.550
Spatial autoregressive	0.838	0.822	0.948	0.547	0.510
Global environmental- autoregressive	0.833	0.818	0.954	0.525	0.480
Local environmental- autoregressive	0.829	0.824	0.961	0.525	0.417

For *A. phagocytophilum*, the AUC for the global environmental model was also considerably lower than all other models (Table 3). In contrast to *E. chaffeensis*, the AUC values for both the local environmental and global environmental-autoregressive models were higher than the spatial autoregressive model, and the AUC for the local environmental-autoregressive model was the highest of all the models. The ranking of *A. phagocytophilum *models based on classification accuracy was the same as the ranking based on AUC. Predictions of *A. phagocytophilum *presence/absence had slightly higher specificity than sensitivity for all models except the spatial autoregressive model. The AUC and classification accuracy for *A. phagocytophilum *were always lower than the statistics for the corresponding *E. chaffeensis *models.

**Table 3:** Predictive accuracy of five statistical models for the distribution of *Anaplasma phagocytophilum *in the southeastern and south-central United States.

Model	AUC	Accuracy	Sensitivity	Specificity	Threshold
Global environmental	0.700	0.658	0.592	0.721	0.504
Local environmental	0.756	0.700	0.567	0.828	0.611
Spatial autoregressive	0.748	0.679	0.776	0.586	0.456
Global environmental- autoregressive	0.765	0.704	0.570	0.831	0.581
Local environmental- autoregressive	0.777	0.713	0.621	0.800	0.564

**Figure 5:**
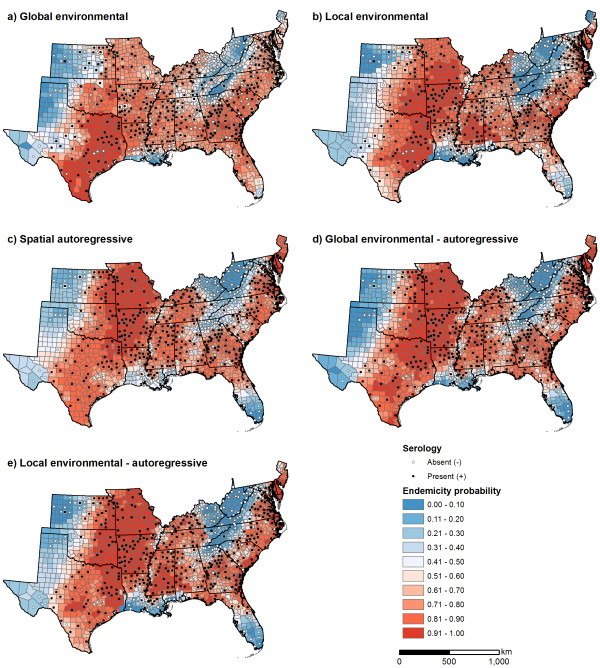
Predicted endemicity probabilities for *Ehrlichia chaffeensis *in the southeastern United States obtained from five Bayesian hierarchical models.

Spatial patterns of predicted endemicity probabilities differed among the models. For *E. chaffeensis*, the local environmental model resulted in improved predictions along the eastern range boundary surrounding the southern Appalachian mountain chain, and along the western range boundary at the transition between the eastern deciduous forest ecoregion and the Great Plains (Figure 5a–b). Incorporating a spatial autoregressive term resulted in further refinements of the eastern and western range boundaries, as well as more accurate predictions of the absence of *E. chaffeensis *in southern Florida and in small pockets along the Mississippi River valley (Figure 5c–e). The models that included environmental variables all produced a more distinct range boundary in central Texas than the spatial autoregressive model.

**Figure 6:**
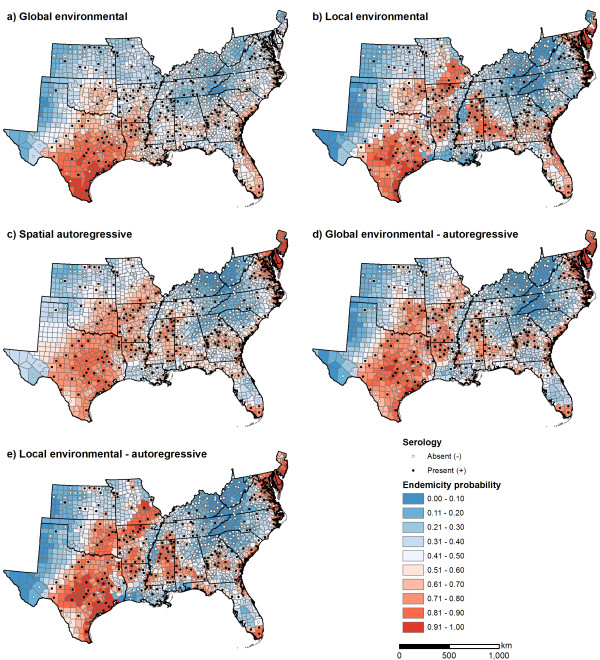
Predicted endemicity probabilities for *Anaplasma phagocytophilum *in the southeastern United States obtained from five Bayesian hierarchical models.

For *A. phagocytophilum*, the local environmental model improved predictions compared to the global environmental model along the Atlantic coast and in the areas surrounding the Mississippi Delta (Figure 6a–b). The spatial autoregressive model resulted in similar improvements, and predicted a more continuous zone of high endemicity ranging from east Texas through Oklahoma, Arkansas, and into southeast Missouri (Figure 6c–e). Patterns predicted by the global environmental-autoregressive and local environmental-autoregressive were similar to those predicted by the spatial autoregressive model. As with *E. chaffeensis*, only the models with a spatial autoregressive component correctly predicted the distribution of *A. phagocytophilum *in Florida, and models that included environmental variables produced a more distinct range boundary in central Texas than the spatial autoregressive model.

## Discussion

Predicted endemicity probabilities based on environmental variables reflect the ecology of the tick vectors and mammalian host communities. Development rates of larval, nymph, and adult ticks increase with temperature [39], and extremely low temperatures can also result in mortality of overwintering eggs [40]. Negative influences of low temperatures on tick populations are reflected in the absence of both *E. chaffeensis *and *A. phagocytophilum *from higher elevations in the southern Appalachian mountain range. However, off-host ticks and eggs are also susceptible to mortality from a combination of high temperature and low atmospheric moisture [41]. *I. scapularis*, the vector of *A. phagocytophilum*, is particularly susceptible to desiccation and typically selects for habitats characterized by forested overstories, high shrub density, deciduous leaf litter, and other structural features that maintain high levels of humidity at the forest floor [42,43]. The relationships between endemicity probability and forest cover in the environmental models likely capture some of these influences of vegetation structure on local microclimates.

In addition to suitable microhabitats, ticks require sufficient populations of mammalian hosts for blood meals. These hosts may also serve as reservoirs for tick-borne pathogens, allowing their transmission to the next generation of uninfected ticks. Because white-tailed deer are hosts for all three life-stages of *A. americanum *[20] and competent reservoirs for *E. chaffeensis *[21], a relatively small deer population is sufficient to support *E. chaffeensis *in a stable enzootic cycle. In contrast, white-tailed deer are effectively a dead-end host for *A. phagocytophilum*. Although white-tailed deer are typically required to maintain high population densities of *I. scapularis *[44], one or more additional host species are necessary to sustain *A. phagocytophilum *in a stable enzootic cycle. Adult *I. scapularis *feed on deer and can either transmit or acquire infection. However, they feed only once at the adult stage and thus cannot spread the pathogen because *A. phagocytophilum *is not transovarially transmitted from adults to larvae. Instead, transmission must be sustained by small mammals that allow *I. scapularis *to acquire infection at the larval stage and transfer it to uninfected hosts at the nymph stage. The importance of host availability is demonstrated by the relationship between *A. phagocytophilum *endemicity probability and deer density. The influences of forest cover and fragmentation on endemicity probability may also reflect indirect effects of habitat suitability for mammalian host species [45-47].

The ecological differences between *E. chaffeensis *and *A. phagocytophilum *are manifested in their geographic distributions. *E. chaffeensis *is transmitted by a tick species with broad environmental tolerance and requires only a single host species that is common across most of the southeastern and south-central United States. Because of these characteristics, it is endemic across most of its range and has a fairly continuous distribution within its external range boundaries. In contrast, *A. phagocytophilum *is transmitted by a tick species that is more sensitive to environmental extremes and requires one or more additional host species besides white-tailed deer. Compared to *E. chaffeensis*, the lower prevalence and spatially variable distribution of *A. phagocytophilum *likely arise from greater sensitivity to the environmental factors influencing vector populations and host communities.

For both *E. chaffeensis *and *A. phagocytophilum*, the spatial autoregressive model had a higher AUC than the global environmental model. The autoregressive term captures either spatially structured environmental relationships that were not measured by our particular set of environmental variables or spatial processes such as dispersal that can create patterns that are unrelated to the environment. In the case of *E. chaffeensis*, combining environmental variables with the autoregressive term in the global environmental-autoregressive model failed to improve predictions compared to the purely autoregressive model. This finding reflects the highly autocorrelated distribution of *E. chaffeensis*, and demonstrates that information about *E. chaffeensis *presence in neighbouring counties is sufficient to capture all the variability predicted by the climate and land cover variables. In a previous study, we similarly found that spatial interpolation of *E. chaffeensis *based on indicator kriging was more accurate than environmental predictions based on logistic regression models [13]. The conditional autoregressive approach used in this study represents an improvement over indicator kriging because it uses a more natural definition of county neighbourhoods based on adjacency rather than distance, and therefore does not require that county locations be approximated as centroids.

In contrast to *E. chaffeensis*, the combined environmental-autoregressive models had the highest accuracies for *A. phagocytophilum*. The lower accuracy of the purely autoregressive model reflected the relatively weak spatial pattern of *A. phagocytophilum*, which limited the extent to which endemicity could be predicted based on pathogen presence or absence in neighbouring counties. The effectiveness of autoregressive models will similarly be reduced in situations where sample size is low or sample locations are highly clustered, thereby reducing the number of nearby points that are available to support predictions at unsampled locations [48]. The most accurate model for *A. phagocytophilum *was the local environmental-autoregressive model that incorporated spatial variability in the regression coefficients for the environmental variables. The better performance of the local environmental models for this pathogen reflected the higher environmental sensitivity of *I. scapularis *combined with geographic variability in the host species that served as the hosts for *I. scapularis *and as reservoirs for *A. phagocytophilum*. The spatially varying regression coefficients allowed the environmental models to be more closely calibrated to different environmental relationships within each geographic zone.

Besides improving prediction accuracy, spatial heterogeneity can also provide insights into the underlying ecological processes controlling the distributions of zoonotic pathogens. Spatial variability in environmental relationships may reflect genetic variability in pathogens, vectors, or hosts that leads to dominance by different genotypes in different areas [7]. Alternatively, spatial heterogeneity may arise from the coarse nature of the environmental variables used to develop the models [18]. Interpolated climate surfaces, land cover maps, and other geospatial datasets serve as correlates of the microhabitats that are the proximal influences on vector and host populations. Furthermore, tick-borne pathogens are maintained by complex interactions among vector and host species that are not necessarily predictable based solely on habitat associations [49]. In some situations, pathogens have multiple vectors and hosts and can be maintained by different sets of species within different portions of their geographic ranges. Thus, spatial variability in the relationships between zoonotic pathogens and environmental variables can reflect unique ecological situations in different ecoregions. For both *E. chaffeensis *and *A. phagocytophilum*, the availability of microhabitat niches for ticks and mammalian hosts appears to be influenced by climatic gradients in the southeastern U.S. and by variability in land use and land cover in the south-central U.S. [18].

A challenge in developing spatially heterogeneous models such as the ones used in this study is the need to specify geographic zones for the local analysis. One approach is to use an existing ecological stratification such as the ecoregion maps developed by the U.S. Environmental Protection Agency [50]. Alternately, ecoregion boundaries can be delineated through multivariate cluster analysis of climate and other environmental variables [7,51]. In the present study, zones for local modelling were previously created via *k*-means clustering of the results of a geographically weighted regression analysis of pathogen distributions [18]. The advantage of this approach is that the zones are objectively delineated based on the actual relationships between pathogens and environmental variables. However, different zonations will be obtained depending on the types of clustering methods used and the number of clusters selected, and the particular zones used in this study are not necessarily optimal for modelling spatial heterogeneity. Comparison of different methods for geographic stratification was beyond the scope of this study, but would be a valuable area for future research. To avoid the problem of zonation, an alternative approach could be to apply a Bayesian version of geographically weighted regression in which spatial variability in the *b *coefficients is modelled as a spatially autocorrelated random effect [52]. However, this type of model has yet to be applied in a predictive framework.

## Conclusion

Predictive modelling of disease risk can be enhanced using spatially explicit methods that account for either spatial autocorrelation (the tendency for pathogen distributions to be clustered in space) or spatial heterogeneity (the potential for environmental influences on pathogens to vary predictably in space). However, the modelling approach that is most effective will depend on the ecology of the underlying zoonotic cycle and the spatial pattern of the resulting pathogen distributions. For pathogens such as *E. chaffeensis *that have relatively simple zoonotic cycles and are common within the boundaries of their geographic ranges, predictions based on spatial autocorrelation can be very effective when key environmental variables are unknown or unavailable as geospatial datasets. For pathogens such as *A. phagocytophilum *that have multiple hosts and comparatively weak spatial patterns, models that incorporate spatial heterogeneity can improve predictions by capturing geographic shifts in the predominant ecological drivers.

## Competing interests

The authors declare that they have no competing interests.

## Authors' contributions

MCW designed the study, carried out the statistical analysis, and was the lead writer of the manuscript. ADB was responsible for development and management of the geospatial databases and contributed to the writing of the manuscript. MJY contributed to the development of the study, the interpretation of the statistical results, and the writing of the manuscript.

## Supplementary Material

Additional file 1 Parameter Estimates. Mean posterior parameters values from the Bayesian hierarchical models with 2.5% and 97.5% Bayesian credible intervals. Click here for file
