# Supplementary material for: Enhanced spatial models for predicting the geographic distributions of tick-borne pathogens
Source: Int J Health Geogr. 2008 Apr 15;7:15. doi: 10.1186/1476-072X-7-15 (PMC2373776; doi:10.1186/1476-072X-7-15)
Supplement: Additional file 1 — Parameter Estimates. Mean posterior parameters values from the Bayesian hierarchical models with 2.5% and 97.5% Bayesian credible intervals. [file 1476-072X-7-15-S1.pdf]

Additional file 1: Mean posterior parameters values from the Bayesian hierarchical models with 2.5% and 97.5% Bayesian credible intervals.

Table A1: Parameter estimates from the global environmental models.

	Ehrlichia chaffeensis			*Anaplasma phagocytophilum*		
Parameter	Mean	2.5%	97.5%	Mean	2.5%	97.5%
b_0	0.793	0.585	1.004	-0.192	-0.383	-0.003
b_1	1.170	0.877	1.478	0.542	0.313	0.781
b_2	0.519	0.206	0.844	0.856	0.558	1.163
b_3	-1.109	-1.556	-0.670	-0.833	-1.199	-0.475
b_4	1.351	0.946	1.774	0.597	0.283	0.915
b_5	1.094	0.800	1.398	0.266	0.066	0.469

Slope coefficients are listed as b_k. The values of j index the environmental variables (0=intercept, 1=temperature, 2=humidity, 3=precipitation, 4=forest cover, 5=forest fragmentation for *E. chaffeensis* and deer density for *A. phagocytophilum*).

Table A2: parameter estimates from the local environmental models.

	Ehrlichia chaffeensis			*Anaplasma phagocytophilum*		
Parameter	Mean	2.5%	97.5%	Mean	2.5%	97.5%
b_{00}	1.922	0.142	3.786	0.482	-0.333	1.309
b_{10}	2.006	1.362	2.733	0.629	-0.033	1.408
b_{20}	2.423	1.284	3.632	2.534	1.563	3.687
b_{30}	-2.656	-4.318	-1.231	-3.461	-6.308	-1.075
b_{40}	1.053	-1.750	3.788	-0.028	-1.169	1.118
b_{50}	-0.158	-2.025	1.726	0.077	-0.454	0.608
b_{01}	1.566	-0.877	4.225	-0.100	-1.111	0.937
b_{11}	-1.872	-3.215	-0.559	-0.427	-1.358	0.430
b_{21}	-2.223	-3.668	-0.797	-1.668	-2.973	-0.501
b_{31}	3.401	0.971	6.040	2.839	0.276	5.838
b_{41}	0.127	-3.200	3.690	0.611	-0.856	2.075
b_{51}	0.244	-1.939	2.475	0.541	-0.215	1.306
b_{02}	4.312	-0.903	9.834	1.160	-0.095	2.475
b_{12}	-4.016	-8.934	0.648	-1.540	-3.109	-0.077
b_{22}	-5.333	-7.790	-3.208	-2.832	-4.257	-1.532
b_{32}	2.289	-0.233	4.857	2.375	-0.257	5.326
b_{42}	-2.012	-5.222	1.310	0.498	-0.855	1.835
b_{52}	-0.010	-2.371	2.426	0.633	-0.052	1.345
b_{03}	-1.028	-2.948	0.811	-0.651	-1.782	0.490
b_{13}	-1.834	-2.888	-0.827	0.730	-0.370	1.816
b_{23}	-1.970	-3.320	-0.650	-2.007	-4.255	0.192
b_{33}	1.619	-0.120	3.493	3.129	0.438	6.182
b_{43}	0.658	-2.206	3.653	0.055	-1.534	1.621
b_{53}	1.295	-0.675	3.290	0.018	-0.777	0.809

Slope coefficients are listed as b_{jk}. The values of j index the environmental variables (0=intercept, 1=temperature, 2=humidity, 3=precipitation, 4=forest cover, 5=forest fragmentation for *E. chaffeensis* and deer density for *A. phagocytophilum*). The values of k index the geographic zones in Figure 3 (0=zone 1, 1=zone 2, 2=zone 3, and 3=zone 4).

Table A3: Parameter estimates from the spatial autoregressive models.

	Ehrlichia chaffeensis			*Anaplasma phagocytophilum*		
Parameter	Mean	2.5%	97.5%	Mean	2.5%	97.5%
b_0	1.363	0.997	1.760	-0.048	-0.337	0.241
σ^2_ρ	10.250	6.835	14.230	6.798	3.398	10.870

b_0 is the intercept and σ^2_ρ is the variance of the spatial random effect.

Table A4: Parameter estimates from the global environmental/autoregressive models.

	Ehrlichia chaffeensis			*Anaplasma phagocytophilum*		
Parameter	Mean	2.5%	97.5%	Mean	2.5%	97.5%
b_0	1.259	0.871	1.685	-0.265	-0.566	0.035
b_1	1.283	0.400	2.174	0.942	0.239	1.755
b_2	0.751	0.095	1.424	1.050	0.499	1.662
b_3	-1.001	-2.114	0.154	-0.736	-1.690	0.174
b_4	2.087	1.208	2.990	0.941	0.370	1.536
b_5	1.000	0.388	1.648	0.383	0.065	0.718
σ^2_ρ	8.932	5.142	13.260	5.933	2.743	10.180

Slope coefficients are listed as b_k. The values of j index the environmental variables (0=intercept, 1=temperature, 2=humidity, 3=precipitation, 4=forest cover, 5=forest fragmentation for *E. chaffeensis* and deer density for *A. phagocytophilum*). σ^2_ρ is the variance of the spatial random effect.

Table A5: Parameter estimates from the local environmental/autoregressive models.

	Ehrlichia chaffeensis			*Anaplasma phagocytophilum*		
Parameter	Mean	2.5%	97.5%	Mean	2.5%	97.5%
b_{00}	2.255	-0.431	5.065	0.012	-1.913	1.745
b_{10}	2.204	1.091	3.525	1.060	0.006	2.209
b_{20}	2.240	0.771	3.859	2.657	1.380	4.068
b_{30}	-1.880	-4.301	0.369	-2.065	-5.306	1.234
b_{40}	1.418	-1.964	4.894	0.264	-1.296	1.788
b_{50}	0.041	-2.272	2.414	0.156	-0.570	0.887
b_{01}	1.670	-2.419	5.994	1.331	-1.030	3.829
b_{11}	-1.939	-3.994	0.051	-1.027	-2.648	0.540
b_{21}	-2.456	-4.495	-0.478	-1.475	-3.128	0.175
b_{31}	4.176	0.158	7.834	1.834	-1.879	5.312
b_{41}	-0.506	-4.696	3.587	0.592	-1.395	2.638
b_{51}	-0.334	-2.996	2.407	0.482	-0.539	1.533
b_{02}	9.035	0.827	18.270	2.924	0.188	6.011
b_{12}	-8.979	-17.790	-0.993	-2.517	-4.876	-0.212
b_{22}	-5.996	-9.447	-3.035	-3.369	-5.267	-1.586
b_{32}	1.014	-3.093	4.945	0.377	-3.527	4.023
b_{42}	-1.960	-6.286	2.316	0.190	-1.675	2.075
b_{52}	0.276	-2.806	3.372	0.633	-0.290	1.565
b_{03}	-0.507	-3.528	2.401	0.409	-1.749	2.705
b_{13}	-1.752	-3.515	-0.061	0.895	-0.737	2.626
b_{23}	-1.228	-3.237	0.644	-1.925	-4.850	0.987
b_{33}	1.106	-1.766	3.942	1.442	-2.484	5.124
b_{43}	0.815	-2.961	4.507	-0.114	-2.160	1.970
b_{53}	0.896	-1.685	3.408	-0.229	-1.328	0.840
σ^2_ρ	5.508	3.211	8.180	5.028	2.213	8.292

Slope coefficients are listed as b_{jk}. The values of j index the environmental variables (0=intercept, 1=temperature, 2=humidity, 3=precipitation, 4=forest cover, 5=forest fragmentation for *E. chaffeensis* and deer density for *A. phagocytophilum*). The values of k index the geographic zones in Figure 3 (0=zone 1, 1=zone 2, 2=zone 3, and 3=zone 4). σ^2_ρ is the variance of the spatial random effect.
